# Diseases of the West—their Complications, &c.

**Published:** 1849-01

**Authors:** W. Matthews

**Affiliations:** Eberle, Ind.


### ARTICLE V.

*Diseases of the West—their Complications, &c.* By W. MATTHEWS, M. D., of Eberle, Ind.

It is an erroneous doctrine, first promulged by a high authority, which asserts that but one disease can prey upon the human body at one and the same time. If any Physician has imbibed such a theory, the experience of a short practice in the West would surely explode it.

In confined and illy ventillated Hospitals we know that every ailment, however trivial, is likely to become complicated, and partake of the nature of the pestilential and putrid disorder prevailing in the house at the iime. So, likewise, it is rational to conclude, are most diseases of whatever nature which spring up, so to speak, in the midst of, and surrounded by malarial influence. Even the pure inflammations, it cannot be doubted, are very greatly modified in consequence of the peculiarities of the atmosphere by which they are surrounded. It is a fact notorious to everyobserving practitioner of the West, that, during the autumnal months, puerperal women are extremely obnoxious to paroxysmal peritonitis, requiring, in most instances, for its relief, a remedy scarcely ever mentioned by the writers on puerperal fever. That remedy is quinine in anti-periodic doses. Again, Western practitioners meet, habitually, during the prevalence of our endemic fevers, with cases of acute pneumonic disease which an anti-phlogistic plan of treatment *alone* is inadequate to relieve, but which a few timely doses of quinine will promptly arrest. The cases here referred to I have oftener met with in children than adults.

To more fully illustrate this subject, and put the inexperienced Physician upon his guard, pertaining to the treatment of *our* inflammations, I propose introducing the history of a case or two that came under my management in the Autumn of 1847.

Mr. J., a young man of excellent constitution, came on a visit from an adjoining county to his father's, who resides near me. Previous to making his visit, J. had complained for some days of *sore eyes*, for the relief of which a practitioner had prescribed an astringent collyrium which, however, had no good effect. I was consulted, and upon inspection, found the case to be one of purulent opthalunia of a severe character, producing very marked constitutional disturbances. He was freely bled, opening medicines ordered and nit. silver directed as a local wash. This treatment checked the disease's progress, but did not arrest it. Again and again, the unpleasant symptoms recurred; and again and again, they were palliated by anti-phlogistics, sedatives, blisters, etc.; the patient the meanwhile remaining in utter darkness. About this time his mother contracted the affection; and soon thereafter, a sister's eyes were also seen to be on fire. Now this was, as will be readily inferred, something of a time of darkness; for I confess that I was groping my way in the dark, and without hesitancy, the patients were willing to confess themselves painfully in the dark. Well, the old lady was bled, purged, blistered, &c.; but with no other good effect than temporary relief—often procured only by opiates. All the symptoms were apt to suffer aggravation at night, and this circumstance led me to suspect a periodic, or, in other words, a *malarial* compication, by which the affection was fanned, so to speak, into a flame, thus, in a great measure, undoing in the course of a few hours, by each returning paroxysm, all that had been gained by anti-plogistics, &c. With a view, therefore, of driving from the system the *supposed cause* [malaria] of the difficulty, quinine in free doses, in union with pill, hydrarg, and morphine was prescribed. And the good effects were as prompt and manifest as those usually following the administration of the same drug in pure, undisguised intermittent. The redness, pain, and swelling of the eyes rapidly, and now permanently disappeared; and in the course of a few days the patients were free from every unpleasant symptom.

Now it is not pretended that the treatment in these cases materially different from that which most Western practitioners would have given them. But suppose an inexperienced student of one of the Eastern Colleges had been called on to manage them? Would he have been apt to prescribe quinine as an antidote? It is true, in the latter stage of the disease, and perhaps, after the local destruction had been complete and permanent, he would have prescribed it as *a tonic*, in one grain doses. But such doses, so far from palliating the local symptoms would most probably aggravate them by imparting tone to the system, and at the same time failing to bring it under the medicine's sedative influence. Hence the great, the indispensible, necessity of studying diseases as they occur in practice—not, as some tell us they occur, alike under all circumstances and in all localities.

Medical students should, in my opinion, study disease where such cases as they will have to treat are exhibited.

There are, indeed, but few diseases that do not undergo modification in passing from one country or locality to another. And these modifications are, not unfrequently, of great consequence to be understood, and correctly cared for. For example: A puerperal woman gets a peritonitis at a time that malaria is exerting its influence upon her system ; she is correctly treated for the peritonitis, but at the moment the inflammation is subsiding, the malaria brings up a paroxysm of fever, which could not fail to re-light the stifled original inflammation. The general excitement subsides slightly, but the local inflammation is slightly elevated. Another paroxysm comes on, and with it comes up, to a point incompatible with the life of the patient, the local inflammation, and the patient dies, not properly of the peritonitis, but of the malaria by which it was fed. A few doses of quinine, in this case, while they would not by any means have increased the peritoneal inflammation, would most certainly have expelled from the system the malaria by which it was fanned, and the patient would not have died.

